# Glenoid rim morphology in young athletes with unstable painful shoulders: primarily painful vs. frankly unstable

**DOI:** 10.1016/j.jseint.2023.04.004

**Published:** 2023-05-13

**Authors:** Shigeto Nakagawa, Kunihiko Hiramatsu, Yuzo Yamada, Kenji Yoneda, Yoshinari Tanaka, Yukiyoshi Toritsuka, Tatsuo Mae

**Affiliations:** a Department of Orthopaedic Sports Medicine, Yukioka Hospital, Osaka, Osaka, Japan; b Department of Orthopaedic Surgery, Tamai Hospital, Hannan, Osaka, Japan; c Department of Orthopaedic Surgery, Yao Municipal Hospital, Yao, Osaka, Japan; d Yoneda Sports Orthopedics, Suita, Osaka, Japan; e Osaka Metropolitan University, Graduate School of Human Life and Ecology, Habikino, Osaka, Japan; f Mukogawa Woman’s University, Nishinomiya, Hyogo, Japan; g Yukioka Medical University, Ibaraki, Osaka, Japan

**Keywords:** Unstable painful shoulder, Glenoid rim morphology, Unrecognized anteroinferior instability, Three-dimensional computed tomography, Collision/contact athlete, Unstable shoulder, Bone fragment

## Abstract

**Background:**

To investigate the characteristics of glenoid rim morphology in young athletes (<40 yr) with unstable painful shoulder

**Methods:**

This was a retrospective case series. The inclusion criteria were as follows: (1) shoulder pain during sports activity, (2) traumatic onset, (3) no complaint of shoulder instability, and (4) soft tissue or bony lesions confirmed on imaging examinations (computed tomography and magnetic resonance imaging). The above-mentioned painful cohort was then compared (in a 2:1 ratio) to a match-paired control group of patients with similar demographics but with frank anterior glenohumeral instability as defined by imaging and physical findings. The pain (not apprehension) was reproduced during the anterior apprehension test in supine position and relieved by relocation test in all patients. Glenoid rim morphology, bone union in shoulders with a fragment-type glenoid, glenoid defect size, bone fragment size, medial displacement of bone fragments (MDBF), and medial distance of erosion (MDE) were compared between painful shoulders and unstable shoulders.

**Results:**

There were 79 painful shoulders and 165 unstable shoulders. The glenoid rim morphology was normal in 33 shoulders, erosion-type in 15 shoulders, and fragment-type in 31 shoulders among painful shoulders, whereas the respective shoulders were 19, 33, and 113 among unstable shoulders (*P* < .001). Bone union was complete in 15 shoulders, partial in 14 shoulders, and nonunion in 2 shoulders among painful shoulders, whereas the respective shoulders were 43, 31, and 39 among unstable shoulders (*P* = .001). The mean glenoid defect size was 6.0 ± 7.2% and 12.7 ± 7.4%, respectively (*P* < .001), and the mean bone fragment size was 5.8 ± 6.4% and 5.4 ± 4.6%, respectively, (*P* = .591). The mean MDBF was 1.4 ± 1.5 mm and 3.0 ± 2.2 mm, respectively (*P* < .001), and the mean MDE was 2.3 ± 1.2 mm and 5.2 ± 2.4 mm, respectively (*P* < .001). In shoulders with a smaller glenoid defect (<13.5%), the prevalence of shoulders with MDBF (<2 mm) and shoulders with MDE (<2 mm) was more frequent in painful shoulders. On the other hand, in shoulders with a larger glenoid defect (≥13.5%), erosion-type glenoid, nonunion in fragment-type glenoid and bone fragment smaller than 7.5% was not recognized in painful shoulders. Shoulders with MDBF (<2 mm) were significantly more frequent in painful shoulders (*P* = .009).

**Conclusions:**

In painful shoulders normal or erosion-type glenoid was predominant, and glenoid defect size was significantly smaller than unstable shoulders. On the other hand, a large bone fragment (≥7.5%) remained and united completely or partially in all shoulders with a larger glenoid defect (≥13.5%). Bone union was obtained within 2 mm from the articular surface in most of them.

Young athletes sometimes feel only pain but continue to play without recognizing instability even at recurrent instability. Boileau et al termed such condition as unstable painful shoulder (UPS).^1^ They defined it as shoulder pain related to anteroinferior instability without any apparent history of dislocation or subluxation but with true anatomic (soft tissue or bony) rollover lesion. As their participants were restricted to overhead athletes and they did not include athletes with traumatic onset, many atraumatic hyperlax athletes were included. However, we often experienced collision/contact athletes with painful shoulders after traumatic episode. Recently, Hoshika et al redefined the definitions of UPS and included collision/contact athletes with traumatic onset.^5^ They concluded that there were two different types of pathologies: Bankart lesions in lax shoulders and bony Bankart lesions in collision/contact athletes. However, they did not report precise glenoid rim morphology.

Sugaya et al reported that in patients with recurrent anterior instability, abnormal glenoid morphology could be detected in up to 90% of patients.^19^ Among them, a bony Bankart lesion was found in 50% of the shoulders (fragment-type) and a bone fragment was not recognized in 40% of them despite the presence of a glenoid defect (erosion-type). Therefore, we should recognize that shoulder instability is not always a simple dislocation event; it often involves bony injury to the glenoid rim—either via erosion or bony Bankart fracture. Furthermore, there have been many reports regarding glenoid defect and retained bone fragment of bony Bankart lesion.^2^, ^3^, ^4^^,^^6^, ^7^, ^8^, ^9^^,^^11^, ^12^, ^13^, ^14^, ^15^, ^16^, ^17^, ^18^^,^^20^ In patients with such a glenoid defect and a bone fragment, at recurrent anterior instability the bone fragment sometimes appears small compared with the size of the glenoid defect. As reason for the discrepancy, bone fragment absorption and/or glenoid defect enlargement were reported.^4^^,^^9^^,^^11^^,^^15^^,^^17^ On the other hand, Nakagawa et al recently reported that a large bone fragment frequently remained in shoulders with a large glenoid defect at recurrent instability.^12^^,^^13^ Accordingly, it has been clarified that several glenoid rim morphologies exist in shoulders with a fragment-type glenoid.

The purpose of the present study was to investigate the characteristics of glenoid rim morphology using computed tomography (CT) examination in younger athletes with UPS. We hypothesized that such patients would have glenoid fractures, but that the bone fragments would be large, united, and with minimal displacement.

## Materials and methods

This was a retrospective case series of prospectively collected clinical data. The study was approved by the local Institutional Review Board, and participants gave written informed consent to participate.

UPS was defined as purely painful form of shoulder instability, in which chronic shoulder pain can be related to anteroinferior instability. As our patients did not always undergo stabilization surgery, we modified the criteria by Hoshika et al.^5^ The inclusion criteria of the present study were as follows: (1) shoulder pain during sports activity, (2) traumatic onset, (3) no complaint of shoulder instability, and (4) soft tissue or bony lesions confirmed on imaging examinations (CT and/or magnetic resonance imaging [MRI]). Participants were younger athletes (<40 yrs) who came to our hospital, because they feel only pain but continue to play without recognizing instability. They were confirmed as anteroinferior instability using imaging and physical findings. As the specific physical findings, the pain (not apprehension) was reproduced during the anterior apprehension test in supine position and relieved by relocation test in all patients. Throwing athletes with superior labrum anterior to posterior lesion, internal impingement, and glenohumeral internal rotation deficit, who often showed positive anterior apprehension and relocation test, were excluded using MRI and physical examination. Older patients (≥40 yrs), patients without sports activity, patients without CT examination, and patients that underwent previous shoulder surgery were also excluded from the present study.

As the “unstable shoulders”, a match-paired control group of patients who complained of shoulder instability were selected in a 2:1 ratio with similar demographics (athletes < 40 yrs) but with frank anterior glenohumeral instability as defined by imaging (CT and MRI) and physical findings. While they experienced at least once frank dislocation, the apprehension was reproduced during the anterior apprehension test in supine position and relieved by relocation test in all patients.

CT was usually performed at the first hospital visit. CT scanning and image reconstruction were performed with a whole body scanner (spiral scan, 0.5 mm slice thickness, 0.3 mm reconstruction, and three-dimensional {3D} edit mode). For multiplanar reconstruction (MPR), CT data were analyzed in the Digital Imaging and Communications in Medicine mode with Digital Imaging and Communications in Medicine software.

First, according to the classification by Sugaya et al,^19^ the glenoid morphologic characteristics were divided into three groups: normal, erosion-type in which no bone fragments were found despite the presence of glenoid defects, and fragment-type in which bone fragments and glenoid defects were found. Both 3D-CT images and MPR images were used to evaluate bone union according to the classification by Nakagawa et al.^10^^,^^14^^,^^16^ Continuity between the bone fragment and glenoid in all MPR slices was considered *complete union*; bone union on 3D-CT images but discontinuity in some MPR slices, *partial union*; and a lack of continuity in all MPR slices, *nonunion*.

**Figure 1:**
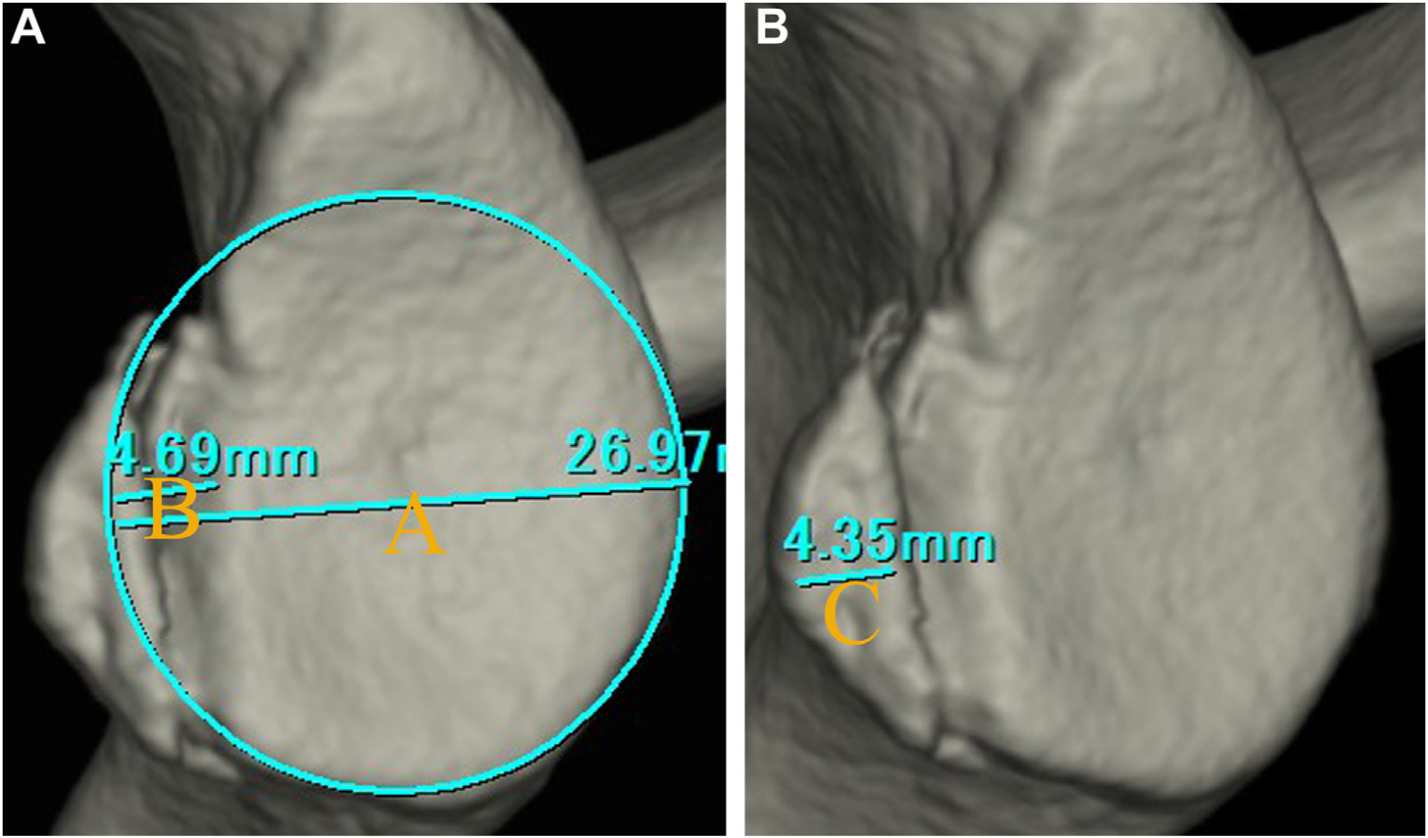
Quantification methods for glenoid defect size and bone fragment size. (**A**): the glenoid defect size: B/A×100%. (A): the diameter of the fitted circle, (B): the glenoid defect width. (**B**): the bone fragment size: C/A × 100%. (C): the bone fragment width.

**Figure 2:**
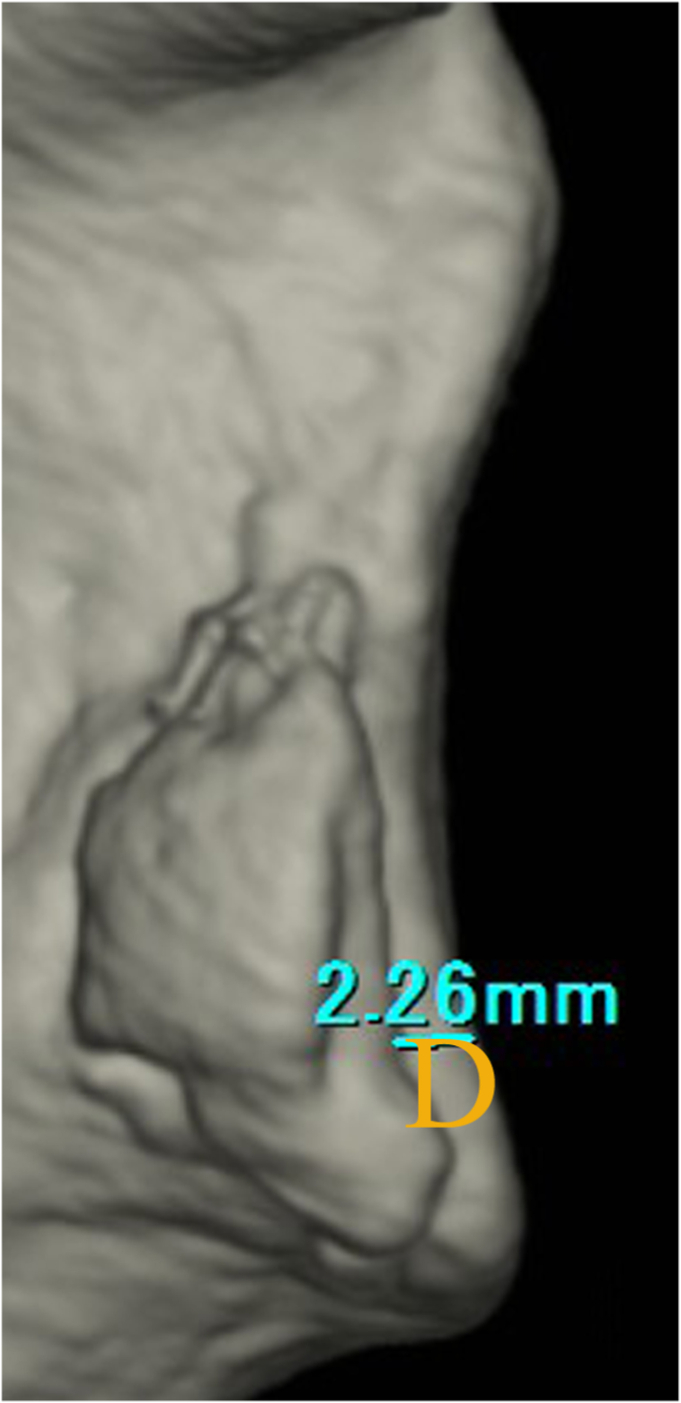
Quantification methods for medial displacement of bone fragments (MDBF). (D): the length of medial displacement of bone fragments from the articular surface

**Figure 3:**
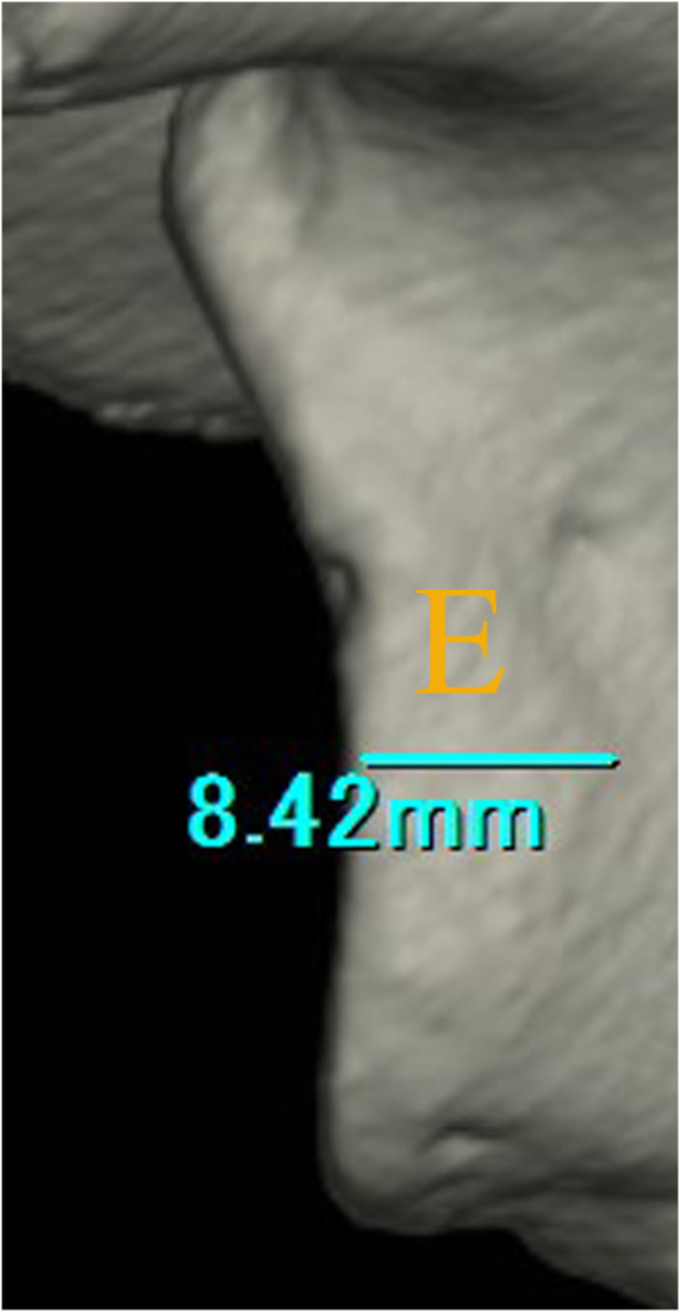
Quantification methods for medial distance of erosion (MDE). (E): the length of medial distance of erosive change from the articular surface.

To quantify the glenoid defect, the inferior portion of the glenoid rim was approximated to a true circle on en face 3D-CT scans that were reconstructed without the head of the humerus. The extent of the glenoid defect was calculated as a percentage of the glenoid rim width (Fig. 1).^10^^,^^14^^,^^16^ The width of the bone fragment was measured on the image that gave the clearest view of the articular surface of the fragment, and the size of the bone fragment was defined relative to the glenoid rim. To examine how much bone fragments were displaced medially from the articular surface, the degree of medial displacement of bone fragments (MDBF) was measured in millimeters by looking at an image in which bone fragments could be viewed from the front of the glenoid fossa (Fig. 2).^10^ Similarly, to examine how much erosive changes enlarged medially from the articular surface, the medial distance of erosion (MDE) was measured in millimeters by looking at an image in which erosive change could be viewed from the front of the glenoid fossa (Fig. 3). To determine the intra- and interobserver reliabilities, we compared 2 intraobserver measurements performed 1 month apart and measurements by 2 independent observers, respectively.

First, features of painful shoulders were investigated, especially noticing glenoid rim morphology, bone union in shoulders with fragment-type glenoid, glenoid defect size, bone fragment size, MDBF, and MDE. Then those factors were compared between painful shoulders and unstable shoulders.

### Statistical analysis

For statistical analysis, mean values of 2 groups were compared with Student *t* test or Mann-Whitney *U* test. In addition, Fisher exact probability test was used to compare the proportions of each factor. When the *P* value < .05, a post hoc power analysis was performed. If the study had sufficient statistical power (1-β ≥ 0.8), we determined the difference to be significant.

To investigate the intra- and interobserver reliabilities, we determined the intraclass correlation coefficient (1, 1) and interclass correlation coefficient (2, 1); a value ≤0.8 was considered good.

## Results

### Demographics

From April 2011 to October 2022, 88 patients, who were compatible with our inclusion criteria, were diagnosed as UPS. Two patients without CT examination, 5 patients of ≥40 yrs, and 2 patients without sports activity were excluded. Accordingly, 79 athletes with UPS, who were <40 yrs and underwent CT examination, were the participants of the present study. Patients profile was shown in Table I.

**TABLE I:** Patients profile.

	Painful (n = 79)	Unstable (n = 165)	*P* value
Gender			
Male	68 (86.1%)	149 (90.3%)	.384
Female	11	16	
Affected side			
Right	48 (60.8%)	76 (46.1%)	.040
Left	31	89	
(Bilateral)	4	8	
Age at computed tomography			
10-19 y	51 (64.6%)	100 (60.1%)	.576
20-29 y	23	60	
30-39 y	5	5	
Age at primary trauma			
10-19 y	53 (69.7%)	133 (80.6%)	.070
20-29 y	19	29	
30-39 y	4	3	
Unknown	3	0	
Period since primary trauma			
<1 y	51 (67.1%)	62 (37.6%)	<.001
1-2 y	14	36	
≥2 y	11 (14.5%)	67 (40.6%)	<.001
unknown	3	0	
Total painful events			
1	49 (64.5%)		
2-5	16		
6-10	6		
≥11	8		
Total instability events			
1		6 (3.6%)	
2-5		67	
6-10		39	
≥11		53	
Sport			
Rugby	32	54	.397 (Collision/contact; 75.9% vs. 81.2%)
American football	12	32	
Contact	16	48	
Overhead	12	19	
Others	7	12	

### Glenoid defect size and bone fragment size in painful shoulders

The correlation between the glenoid defect size (divided into 4 groups; 0%, 0%-10%, 10%-20%, and ≥20%) and the bone fragment size (divided into 4 groups; 0%, 0%-5%, 5%-10%, and ≥10%) was shown in Table II. The largest glenoid defect size in shoulders with an erosion-type glenoid was 12.6%. When the cut-off value of glenoid defect size was set at 13.5%, which was known as the subcritical glenoid bone loss,^18^ the largest bone fragment size was 7.9% in shoulders with a smaller glenoid defect (<13.5%). On the other hand, in shoulders with a larger glenoid defect (≥13.5%), the smallest bone fragment size was 7.5%. Accordingly, we determined the cut-off value of bone fragment size as 7.5%. While in shoulders with a smaller glenoid defect (<13.5%) bone fragment size was 7.5% or larger in 2 shoulders and smaller than 7.5% in 14 shoulders, in shoulders with a larger glenoid defect (≥13.5%) the size was 7.5% or larger in all 15 shoulders.

**TABLE II:** Glenoid defect size and bone fragment size in painful shoulders

		Bone fragment size					Total
		0%	0%-5%	5%-10%		≥10%	
				<7.5%	≥7.5%		
Glenoid defect size							
0%	33						33
0%-10%		13	10	2	2	0	27
10%-20%							
<13.5%		2	0	2	0	0	4
≥13.5%		0	0	0	4	6	10
≥20%		0	0	0	0	5	5
Total	33	15	10	4	6	11	79

### Feature of glenoid rim morphology in painful shoulders: difference between shoulders with a smaller glenoid defect (<13.5%) and shoulders with a larger glenoid defect (≥13.5%)

**Figure 4:**
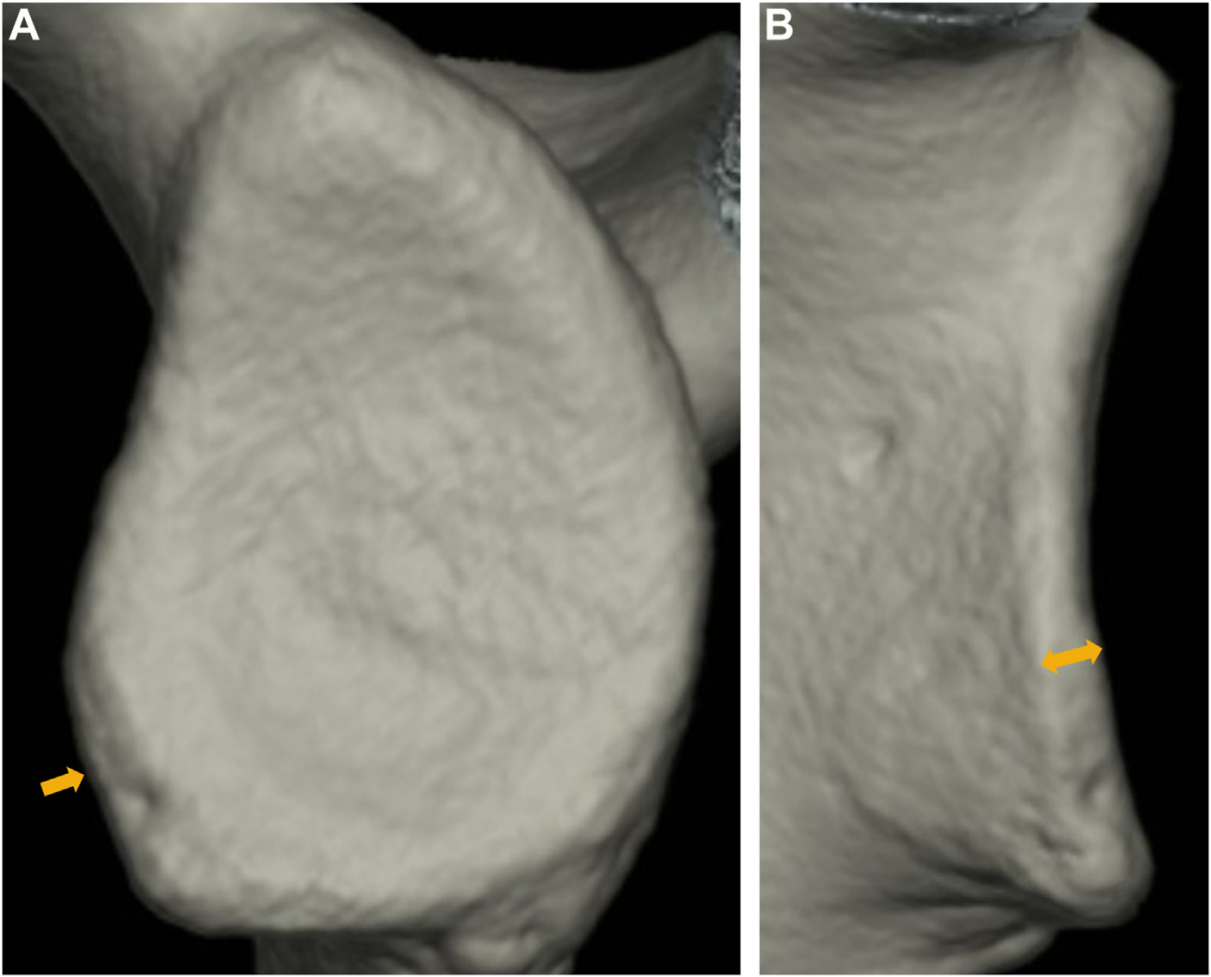
Painful shoulder with a smaller glenoid defect in erosion-type glenoid (25 year-old-male semi-professional American football player). He complained of continued pain at playing football lasting for 6 months after primary traumatic episode. Erosive change was recognized (). (**A**): glenoid defect size; 5.6%. (**B**): medial distance (); 1.3 mm.

**Figure 5:**
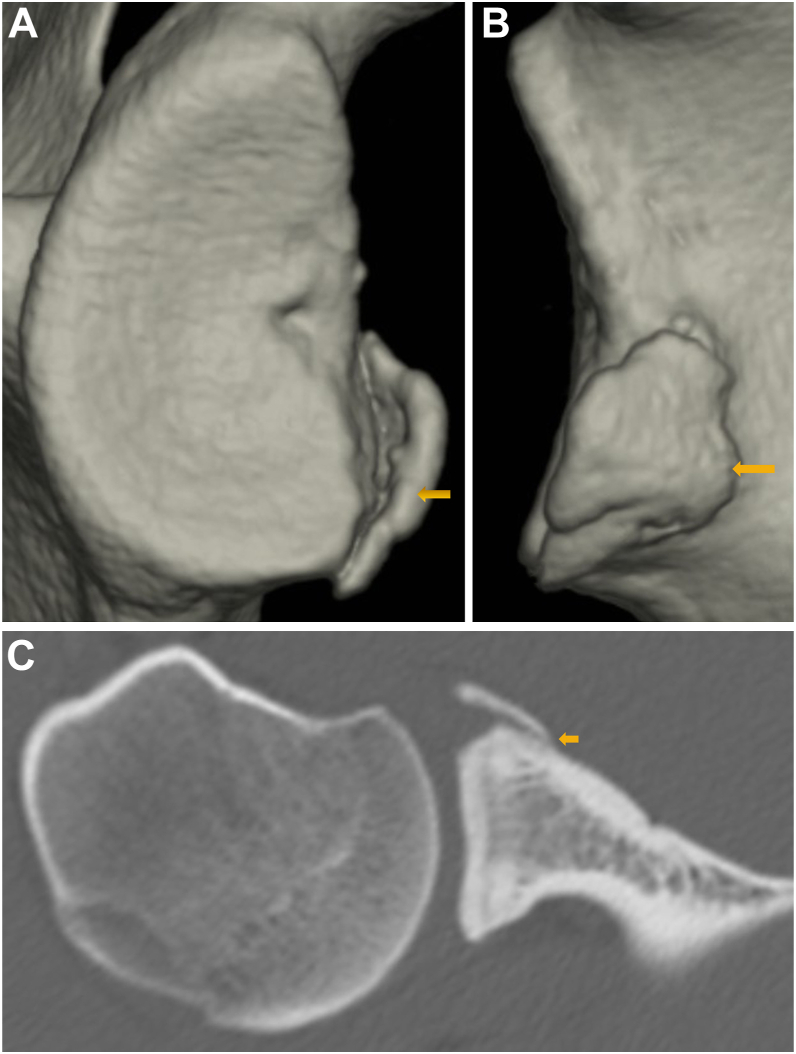
Painful shoulder with a larger glenoid defect accompanying a large bone fragment in fragment-type glenoid (17 year-old-male high school rugby player). He complained of repetitive painful events (120 events) at tackling for 10 months after primary traumatic episode. A large bone fragment was recognized (). (**A**): glenoid defect size; 17.3%, bone fragment size; 9.2%. (**B**): medial displacement; 0 mm. (**C**): partial union.

Glenoid rim morphology in painful shoulders was shown in Table III. They were divided into 2 groups: shoulders with a smaller glenoid defect (<13.5%) and shoulders with a larger glenoid defect (≥13.5%) and feature of glenoid rim morphology was investigated in each defect group. Erosion-type glenoid was solely recognized in shoulders with a smaller glenoid defect (Fig. 4). Among shoulders with a smaller defect, characteristic morphology was not recognized in shoulders with a fragment-type glenoid. On the other hand, nonunion was not recognized in shoulders with a larger glenoid defect. Furthermore, MDBF was <2 mm in 80% of them (Fig. 5).

**TABLE III:** Differences of glenoid rim morphology between shoulders with a smaller glenoid defect and shoulders with a lager glenoid defect among painful shoulders.

	Glenoid defect		*P* value
	<13.5% (n = 31)	≥13.5% (n = 15)	
Glenoid rim morphology			
Erosion	15	0	
Fragment	16	15	<.001
Bone union in fragment-type glenoid			
Complete	10 [5]	5 [3]	
Partial	4 [3]	10 [9]	
Nonunion	2 [0]	0	.046
Bone fragment size			
0%	15	0	
<7.5%	14	0	
≥7.5%	2	15	<.001
MDBF			
<2 mm	8	12	
2 mm-3 mm	3	2	
≥3 mm	5	1	.135
MDE			
<2 mm	8		
2 mm-3 mm	3		
≥3 mm	4		

*MDBF*, medial displacement of bone fragment; *MDE*, medial distance of erosion.

[ ]: MDBF < 2 mm.

The prevalence of shoulders with MDBF (<2 mm) in shoulders with complete or partial union: painful shoulders [8/14 (57.1%)] vs. unstable shoulders [12/15 (80%)]; *P* = .245.

The intraobserver reliabilities for the measurement of glenoid defect size, bone fragment size, MDBF, and MDE were 0.933, 0.923, 0.983, and 0.911, respectively, and the interobserver reliabilities were 0.902, 0.844, 0.901 and 0.874, respectively.

### Difference of glenoid rim morphology between painful shoulders and unstable shoulders

As unstable shoulders, 165 younger athletes (<40 yrs) were selected. Regarding patients profile, as the mean age at CT was 19.1 ± 5.1 yrs in painful shoulders and 19.5 ± 3.9 yrs in unstable shoulders (*P* = .555), and the mean age at primary trauma was 18.2 ± 4.7 yrs in painful shoulders and 17.4 ± 3.3 yrs in unstable shoulders (*P* = .103), there was no difference between them. On the other hand, as the mean period since primary trauma was 0.8 ± 2.1 yrs in painful shoulders and 1.8 ± 2.4 yrs in unstable shoulders (*P* = .002), the period was significantly shorter in painful shoulders. The mean painful events in painful shoulders were 5.8 ± 19.2 and the mean instability events in unstable shoulders were 12.1 ± 17.5 (Table I).

The glenoid rim morphology was normal in 33 shoulders (41.8%), erosion-type in 15 shoulders (19.0%), and fragment-type in 31 shoulders (39.2%) among painful shoulders. On the other hand, the respective shoulders were 19 (11.5%), 33 (20%), and 113 (68.5%) among unstable shoulders. While normal glenoid was significantly more frequent in painful shoulders (*P* < .001), fragment-type glenoid was significantly more frequent in unstable shoulders (*P* < .001) (Table IV).

**TABLE IV:** Differences of glenoid rim morphology between painful shoulders and unstable shoulders.

	Painful (n = 79)	Unstable (n = 165)	*P* value
Glenoid rim morphology			
Normal	33 (41.8%)	19 (11.5%)	<.001
Erosion	15 (19.0%)	33 (20%)	
Fragment	31 (39.2%)	113 (68.5%)	<.001
Bone union in fragment-type glenoid			
Complete	15 (48.4%) [8]	43 (38.1%) [9]	
Partial	14 (45.2%) [12]	31 (27.4%) [12]	
Nonunion	2 (6.5%) [0]	39 (34.5%) [15]	.001
Glenoid defect size			
0%	33 (41.8%)	19 (11.5%)	
<13.5%	31 (39.2%)	82 (49.7%)	
≥13.5%	15 (19.0%)	64 (38.8%)	.002
Bone fragment size			
0%	15 (32.6%)	33 (22.6%)	
<7.5%	14 (30.4%)	71 (48.6%)	
≥7.5%	17 (37.0%)	42 (28.8%)	.360
MDBF			
<2 mm	20 (64.5%)	36 (31.9%)	.002
2 mm-3 mm	5	27	
≥3 mm	6	50	
MDE			
<2 mm	8 (53.3%)	3 (9.1%)	.002
2 mm-3 mm	3	4	
≥3 mm	4	26	

*MDBF*, medial displacement of bone fragment; *MDE*, medial distance of erosion.

[ ]: MDBF < 2 mm.

The prevalence of shoulders with MDBF (<2 mm) in shoulders with complete or partial union: painful shoulders [20/29 (69.0%)] vs. unstable shoulders [21/74 (28.4%)]; *P* < .001.

**Figure 6:**
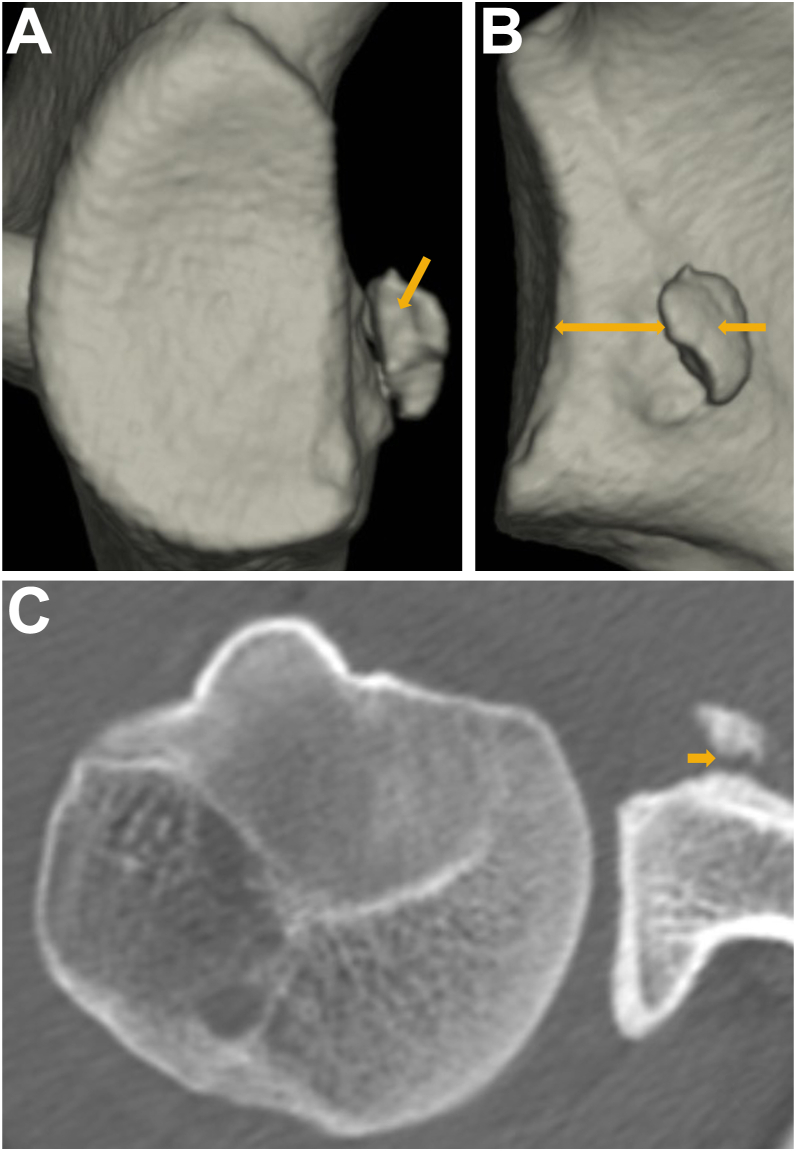
Unstable shoulder with nonunion in fragment-type glenoid (15 year-old-male junior high school rugby player). He complained of repetitive instability events (2 dislocations and 20 subluxations) for 2 years. (**A**): glenoid defect size; 22.0%, bone fragment size; 11.2%. (**B**): medial displacement (); 8.2 mm. (**C**): nonunion. (): bone fragment.

Bone union in fragment-type glenoid was complete in 15 shoulders (48.4%), partial in 14 shoulders (45.2%), and nonunion in 2 shoulders (6.5%) among painful shoulders. On the other hand, the respective shoulders were 43 (38.1%), 31 (27.4%), and 39 (34.5%) among unstable shoulders. Nonunion was significantly less frequent in painful shoulders (*P* = .001) (Fig. 6).

**Figure 7:**
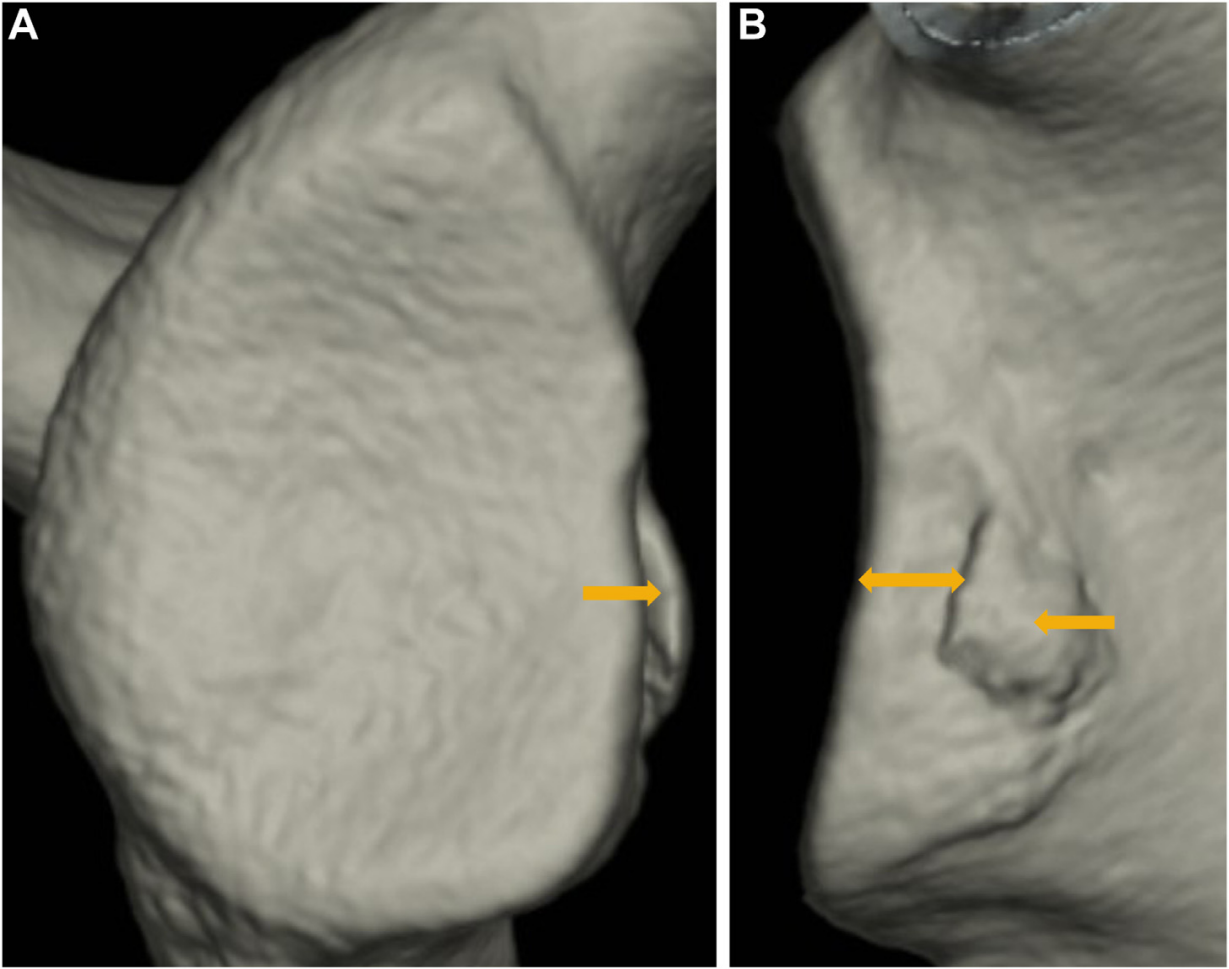
Unstable shoulder with medially displaced bone fragment in fragment-type glenoid (14 year-old-male junior high school rugby player). He complained of repetitive instability events (4 dislocations) for 10 months. (**A**): glenoid defect size; 9.0%, bone fragment size (); 4.9%. (**B**): medial displacement (); 3.8 mm.

**Figure 8:**
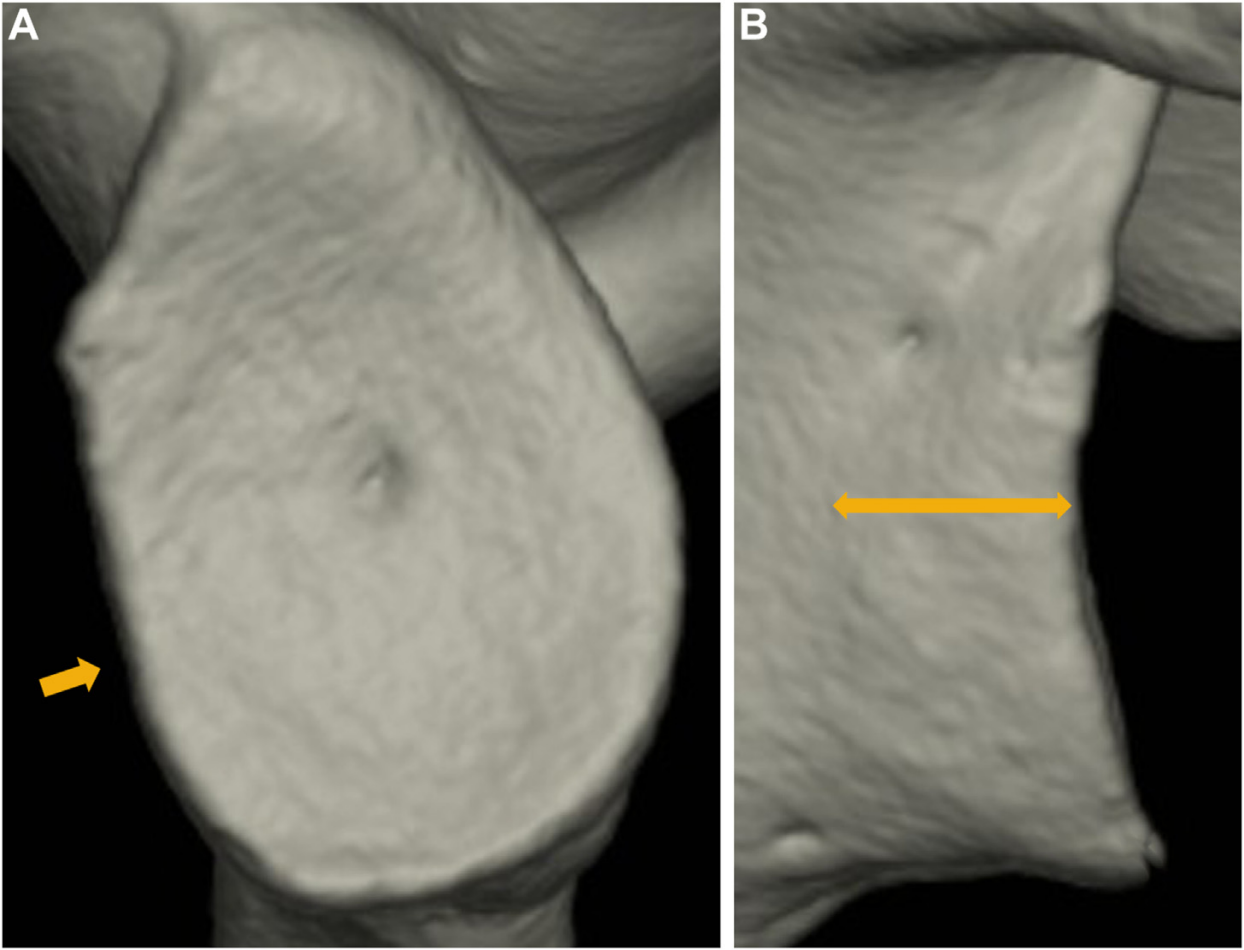
Unstable shoulder with a larger glenoid defect in erosion-type glenoid (18 year-old-male high school rugby player). He complained of repetitive instability events (9 dislocations and 1 subluxation) for 1 year. (**A**): glenoid defect size; 17.2%. (**B**): medial distance (); 7.0 mm. (): erosive change.

As the mean glenoid defect size was 6.0 ± 7.2% in painful shoulders and 12.7 ± 7.4% in unstable shoulders, glenoid defect was significantly smaller in painful shoulders (*P* < .001). On the other hand, in shoulders with a glenoid defect the mean bone fragment size was 5.8 ± 6.4% and 5.4 ± 4.6%, respectively, showing no difference (*P* = .591). As the mean MDBF was 1.4 ± 1.5 mm and 3.0 ± 2.2 mm, respectively, bone fragments did not significantly displace medially in painful shoulders (*P* < .001) (Fig. 7). Furthermore, the prevalence of shoulders with MDBF (<2 mm) in shoulders with complete or partial union was significantly more frequent in painful shoulders (69.0% vs. 28.4%, respectively; *P* < .001). As the mean MDE was 2.3 ± 1.2 mm and 5.2 ± 2.4 mm, respectively, erosive change did not significantly enlarge medially in painful shoulders (*P* < .001) (Fig. 8).

In shoulders with a smaller glenoid defect (<13.5%), the prevalence of shoulders with MDBF (<2 mm) and shoulders with MDE (<2 mm) was more frequent in painful shoulders, whereas no difference was recognized in the other factors (Table V). On the other hand, in shoulders with a larger glenoid defect (≥13.5%), erosion-type glenoid, nonunion in fragment-type glenoid and bone fragment <7.5% was not recognized in painful shoulders (Table VI). Furthermore, shoulders with MDBF (<2 mm) were significantly more frequent in painful shoulders, whereas shoulders with MDE (<2 mm) were not recognized in unstable shoulders. When subjects were restricted to shoulders with a large bone fragment (≥7.5%) among shoulders with a larger glenoid defect (≥13.5%), while the prevalence of shoulders with MDBF (<2 mm) was not different between painful shoulders and unstable shoulders, the prevalence of nonunion was significantly fewer in painful shoulders (*P* < .001) (Table VII).

**TABLE V:** Differences of glenoid rim morphology between painful shoulders and unstable shoulders among shoulders with a smaller glenoid defect (<13.5%).

	Painful	Unstable	*P* value
Glenoid rim morphology			
Erosion	15 (48.4%)	19 (29.7%)	
Fragment	16	45	.109
Bone union in fragment type glenoid			
Complete	10 (62.5%) [5]	24 (53.3%) [2]	
Partial	4 [3]	11 [4]	
Nonunion	2 (12.5%) [0]	10 (22.2%) [2]	.738
Bone fragment size			
<7.5%	14	39	
≥7.5%	2 (12.5%)	6 (13.3%)	.999
MDBF			
<2 mm	8 (50%)	8 (17.8%)	
≥2 mm	8	37	.030
MDE			
<2 mm	8 (53.3%)	3 (15.8%)	
≥2 mm	7	16	.030

*MDBF*, medial displacement of bone fragment; *MDE*, medial distance of erosion.

[ ]: MDBF < 2 mm.

The prevalence of shoulders with MDBF (<2 mm) in shoulders with complete or partial union: painful shoulders [8/14 (57.1%)] vs. unstable shoulders [6/35 (17.1%)]; *P* = .012.

**TABLE VI:** Differences of glenoid rim morphology between painful shoulders and unstable shoulders among shoulders with a larger glenoid defect (≥13.5%).

	Painful	Unstable	*P* value
Glenoid rim morphology			
Erosion	0 (0%)	14 (17.1%)	
Fragment	15	68	.177
Bone union in fragment type glenoid			
Complete	5 (33.3%) [3]	19 (27.9%) [7]	
Partial	10 [9]	20 [8]	
Nonunion	0 (0%)	29 (42.6%) [13]	<.001
Bone fragment size			
<7.5%	0	32	
≥7.5%	15 (100%)	36 (52.9%)	<.001
MDBF			
<2 mm	12 (80%)	28 (41.2%)	
≥2 mm	3	40	.009
MDE			
<2 mm	0	0	
≥2 mm	0	14	

*MDBF*, medial displacement of bone fragment; *MDE*, medial distance of erosion.

[ ]: MDBF < 2 mm.

The prevalence of shoulders with MDBF (<2 mm) in shoulders with complete or partial union: painful shoulders [12/15 (80%)] vs. unstable shoulders [15/39 (38.5%)]; *P* = .014.

**TABLE VII:** Differences of glenoid rim morphology between painful shoulders and unstable shoulders among shoulders with a larger glenoid defect (≥13.5%) and a large bone fragment (≥7.5%).

	Painful	Unstable	*P* value
Bone union in fragment type glenoid			
Complete	5 (33.3%) [3]	7 (19.4%) [5]	
Partial	10 [9]	12 [7]	
Nonunion	0 (0%)	17 (47.2%) [10]	<.001
MDBF			
<2 mm	12 (80%)	22 (61.1%)	.326
≥2 mm	3	14	

*MDBF*, medial displacement of bone fragment.

[ ]: MDBF < 2 mm.

The prevalence of shoulders with MDBF (<2 mm) in shoulders with complete or partial union: painful shoulders [12/15 (80%)] vs. unstable shoulders [12/19 (63.2%)]; *P* = .451.

## Discussion

In painful shoulders normal or erosion-type glenoid was predominant, and glenoid defect size was significantly smaller than unstable shoulders. On the other hand, a large bone fragment (≥7.5%) remained and united completely or partially in all shoulders with a larger glenoid defect (≥13.5%). Bone union was obtained within 2 mm from the articular surface in most of them.

Boileau et al reported that chronic shoulder pain in a young athlete can be related to anteroinferior instability, without any apparent history of dislocation or subluxation, and termed that purely painful form of shoulder instability as UPS.^1^ Its diagnosis is based on the finding of soft tissue or bony lesions, or both, commonly associated with anteroinferior instability and the presence of rollover lesions confirms the history of an unrecognized shoulder subluxation or dislocation. In the present study we investigated glenoid rim morphology in painful shoulders using CT examinations and recognized a glenoid defect at the anteroinferior part of the glenoid fossa in almost 60% of them, which was compatible to assume that this occurred with an unrecognized anteroinferior instability.

We precisely investigated the glenoid defect size and bone fragment size in painful shoulders, and found that the glenoid defect size in shoulders with an erosion-type glenoid was smaller than 13.5%, which was well known as a subcritical glenoid bone loss reported by Shaha et al.^18^ On the other hand, the bone fragment size was ≥7.5% in all shoulders with a larger glenoid defect (≥13.5%). So, in the present study, the features of glenoid rim morphology were individually investigated in shoulders with a larger glenoid defect (≥13.5%) and in shoulders with a smaller glenoid defect (<13.5%), and the size of 7.5% was selected as the cut-off value of the bone fragment size. Interestingly, Nakagawa et al reported that the postoperative recurrence rate after arthroscopic bony Bankart repair was lower in male competitive rugby and American football players with a large glenoid defect (≥13.5%) than in those with a small glenoid defect (<13.5%) and might be associated with a higher rate of complete bone union of the resultant large bone fragment (≥7.5%).^13^ The results of their detailed analysis indicated that a bone fragment size of 7.5% was the appropriate cut-off value for investigating the relationship of bone fragment size with bone union rate and recurrence rate. While the present study was completely different from their study, ≥7.5% might also be the appropriate threshold for a large bone fragment size to investigate the onset of pain or instability.

When the features of glenoid rim morphology were compared with unstable shoulders, in painful shoulders normal glenoid was significantly more frequent and fragment-type glenoid was significantly less frequent. While Boileau et al reported that glenoid osseous lesions were solely recognized in 20% (erosion; 10%, fragment; 10%),^1^ we recognized them in 58.2% and Hoshika et al reported them in 51%.^5^ The proportion (erosion/fragment) was also similar between our result and Hoshika’s report.^5^ While our patients and Hoshika’s patients included collision/contact athletes, Boileau et al restricted their patients to overhead athletes.^1^ The patients profile might influence the difference of glenoid rim morphology.

When the subjects were restricted to shoulders with a smaller glenoid defect (<13.5%), the difference of glenoid rim morphology between painful shoulders and unstable shoulders was not apparent, though the prevalence of shoulders with MDBF (<2 mm) and shoulders with MDE (<2 mm) was significantly more frequent in painful shoulders. Minimal displacement of Bankart lesion (including bony Bankart lesion) seemed to influence the onset of pain.

On the other hand, when the subjects were restricted to shoulders with a larger glenoid defect (≥13.5%), a remaining bone fragment was always large (≥7.5%) and united completely or partially. Furthermore, bone fragment united within 2 mm from the articular surface in 80% of them. Accordingly, bone union with minimal displacement of a large bone fragment appeared to be the characteristic of painful shoulders with a large glenoid defect.

Once the diagnosis was certain, Boileau et al and Hoshika et al reported predictable good results and return to sport after arthroscopic soft-tissue stabilization. So, the appropriate diagnosis of UPS is most important. As the present study clearly showed that CT examination is essential and convenient diagnostic tool, even if the patients do not complain apprehension, CT examination should be performed for patients who complain shoulder pain persisted for a long time after a traumatic episode.

These features in painful shoulders were quite similar to shoulders with an unrecognized glenoid fracture, recently reported by Nakagawa et al.^10^ The authors investigated 38 patients with an unrecognized glenoid fracture in opposite shoulders with symptomatic anterior instability. They had complaints of instability on only one side (symptomatic shoulder), while they had a glenoid fracture despite of no complaint of instability in opposite shoulder (asymptomatic shoulder). They compared them and concluded that shoulders with a completely or partially united bone fragment and with less than 2 mm displacement could be asymptomatic regardless of glenoid defect size. As they also reported that shoulders with a smaller glenoid defect (<10%) could be asymptomatic, even if a bone fragment of bony Bankart was not present, their results regarding small glenoid defects were also similar to us. Accordingly, we could conclude that shoulders with a smaller glenoid defect (<10% or <13.5%) and shoulders with a larger glenoid defect accompanying a large bone fragment with minimal displacement might be asymptomatic or painful.

As the limitation of this study, we solely investigated CT findings. As soft-tissue damage was also the possible cause for the onset of the pain, we would like to investigate MRI findings in the future. Furthermore, while we investigated glenoid rim morphology, noticing bone union of bony Bankart, glenoid defect size, bone fragment size, MDBF and MDE, another bony factor, such as inferior displacement of bone fragments, might induce anteroinferior instability or pain.

## Conclusions

In painful shoulders normal or erosion-type glenoid was predominant, and glenoid defect size was significantly smaller than unstable shoulders. On the other hand, a large bone fragment (≥7.5%) remained and united completely or partially in all shoulders with a larger glenoid defect (≥13.5%). Bone union was obtained within 2 mm from the articular surface in most of them.
